# Integrating Focused Shockwave Therapy into Rehabilitation for Groin Pain Syndrome: A Prospective Study in Soccer Players

**DOI:** 10.3390/life16030509

**Published:** 2026-03-19

**Authors:** Gabriele Santilli, Flavia Santoboni, Elisa Checchi, Antonio Franchitto, Antonello Ciccarelli, Samanta Taurone, Eleonora Latini, Valter Santilli, Giorgio Felzani, Sveva Maria Nusca, Donatella Trischitta, Maria Chiara Vulpiani, Mario Vetrano

**Affiliations:** 1 Department of Movement, Human and Health Sciences, Division of Health Sciences, University of Rome “Foro Italico”, 00135 Rome, Italy; 2 Physical Medicine and Rehabilitation Unit, Sant’Andrea Hospital, Sapienza University of Rome, 00189 Rome, Italy; 3 San Raffaele Sulmona, Viale dell’Agricoltura, 67039 Sulmona, Italy

**Keywords:** focused shockwave therapy, groin pain, athletes, rehabilitation, pilot study

## Abstract

**Background/Objectives**: Groin pain syndrome (GPS) is a frequent and heterogeneous condition in athletes, often associated with persistent pain and functional limitation. Both focused extracorporeal shockwave therapy (f-ESWT) and exercise-based rehabilitation have been proposed as conservative treatment options, but evidence for their combined use in GPS remains limited. This prospective single-arm pilot study aimed to describe temporal changes in pain and function following a multimodal conservative program combining f-ESWT and structured rehabilitation in athletes with GPS, using validated groin-specific outcome measures. **Methods**: Thirty-one consecutive adult soccer players (mean age 28.4 ± 5.8 years; 77.4% male) with clinically and MRI-confirmed GPS underwent three weekly f-ESWT sessions (Duolith; 2400 pulses/session; 4 Hz; energy flux density 0.20 mJ/mm^2^) integrated within a supervised 16-week rehabilitation program (progressive adductor strengthening, core stabilization, and stretching). Outcomes were assessed at baseline (T0), 1 month (T1), and 4 months (T2): HAGOS (primary), VAS pain, and Roles and Maudsley (RM). Within-subject changes were analyzed using repeated-measures ANOVA with Bonferroni correction. **Results**: Statistically significant temporal changes were observed across all outcomes (all *p* < 0.001). HAGOSs changed from 47.23 ± 7.79 at T0 to 77.94 ± 16.18 at T1 and 90.00 ± 14.26 at T2 (partial η^2^ = 0.89). VAS decreased from 6.81 ± 1.25 to 3.68 ± 1.11 and 1.90 ± 1.45 (partial η^2^ = 0.91). RM decreased from 2.39 ± 0.50 to 1.52 ± 0.57 and 1.26 ± 0.63 (partial η^2^ = 0.72). No adverse events were reported. **Conclusions**: In this single-arm pilot study, the multimodal program combining f-ESWT and structured rehabilitation was associated with temporal changes in groin-specific function and pain that exceeded established MCID thresholds. However, in the absence of a control group, these findings are purely descriptive and hypothesis-generating. The observed changes cannot be attributed to f-ESWT specifically, as the 16-week rehabilitation program likely contributed substantially to the outcomes. These preliminary observations require confirmation through adequately powered randomized controlled trials comparing the combined intervention to rehabilitation alone.

## 1. Introduction

Groin pain syndrome (GPS), commonly referred to as “pubalgia”, is a frequent cause of groin pain in athletes, particularly in sports characterized by rapid accelerations, sudden changes of direction, kicking actions, and lateral movements, and sports such as soccer, ice hockey, football, and rugby are among the most frequently reported in the literature, both at professional and non-professional levels [1,2]. Groin pain in athletes is widely prevalent, accounting for approximately 5–13% of injuries in male soccer players and 4–5% in female soccer players; however, its clinical presentation is often heterogeneous and difficult to accurately localize and diagnose [3,4,5].

According to the definition approved at the Italian Consensus Conference on terminology, clinical evaluation, and imaging for groin pain in athletes, GPS is defined as “any clinical symptom located in the inguinal–pubic–adductor region that impacts sports activities and/or interferes with daily life activities, necessitating medical intervention” [6].

From a clinical perspective, recommended assessment protocols for GPS include specific evaluations targeting different anatomical regions. In athletes with suspected adductor-related pain, assessment typically involves palpation of the pubic insertion at the common rectus abdominis/adductor longus aponeurosis, isometric squeeze tests [3,7,8,9,10,11,12,13].

Osteitis pubis represents one of the most common causes of GPS in athletes, with reported incidences ranging from 0.5% to 8%, particularly among distance runners and male soccer players, in whom it accounts for approximately 10–18% of injuries per year [14,15]. Although its exact etiology remains incompletely understood, muscle imbalance between the abdominal and hip adductor muscles is considered a major contributing factor in the development of pubic symphysis overload and inflammation [16]. Imaging techniques such as radiographs, triple-phase bone scintigraphy, and magnetic resonance imaging are commonly used to support diagnosis and to exclude alternative causes of groin pain [17]. In this diagnostic framework, musculoskeletal ultrasound has gained increasing relevance as a complementary tool to MRI, allowing dynamic assessment, side-to-side comparison, and real-time evaluation of superficial soft tissues, while MRI remains essential for comprehensive visualization of deep structures and bone-related abnormalities [18,19,20].

Conservative treatment is widely recommended as the first-line approach for groin pain and related conditions. Commonly adopted strategies include rest and modification of sports activities [21,22], oral medications such as non-steroidal anti-inflammatory drugs [23], and structured rehabilitation programs combining manual therapy, cryotherapy, therapeutic exercises, and stretching [24,25]. Additional non-surgical treatment options described in the literature include local corticosteroid injections [25,26], Botulinum Toxin [27,28], prolotherapy [29], the use of compression garments [30], intra-tissue percutaneous electrolysis [31], and pulse-dose radiofrequency therapy [32].

In recent years, focused extracorporeal shockwave therapy (f-ESWT) has emerged as a potentially effective treatment option for groin pain, with preliminary studies reporting reductions in pain and improvements in function [33,34].

However, the current evidence is limited by the incompleteness of the outcome measures analyzed. In particular, the only randomized controlled trial currently available did not include validated, groin-specific patient-reported outcome measures such as the Copenhagen Hip and Groin Outcome Score (HAGOS), nor global clinical outcome measures assessing perceived pain and functional limitation, such as the Roles and Maudsley score, thereby limiting the interpretation of treatment effectiveness from a patient-centered perspective.

This prospective single-arm pilot study aimed to describe short- and medium-term temporal changes in pain and function following f-ESWT combined with a standardized rehabilitation program in athletes with GPS, using validated, groin-specific patient-reported outcome measures. Given the absence of a control group, this study was explicitly designed as a preliminary, hypothesis-generating investigation to inform the design of future controlled trials.

We hypothesized that a multimodal conservative program combining f-ESWT and structured rehabilitation would be associated with temporal improvements in groin-specific function and pain over 4 months; that observed changes would exceed established minimal clinically important difference (MCID) thresholds; and that the intervention would be well tolerated with no serious adverse events.

## 2. Materials and Methods

### 2.1. Ethical Approval and Consent to Participate

The study was conducted in accordance with the principles of the Declaration of Helsinki and received prior approval from the Institutional Ethics Committee of our institution, Sapienza University’s Institutional Review Board (Prot. 0000/2024—Approval Date: 14 February 2024). Patients were not involved in planning the study design. All patients were enrolled in the study after taking written informed consent. The work of this study is being reported as per the STROBE criteria.

### 2.2. Study Design and Population

This was a prospective, single-arm, single-center pilot study conducted at the Physical Medicine and Rehabilitation Unit of Sant’Andrea University Hospital, Rome, Italy, between November 2022 and October 2024. The single-arm design was chosen as an initial exploratory step to evaluate the feasibility, safety, and preliminary clinical outcomes of the combined intervention before investing resources in a controlled trial. We acknowledge that this design precludes causal inference regarding treatment effectiveness, as observed changes may reflect natural history, regression to the mean, placebo effects, or the effects of the concurrent rehabilitation program rather than f-ESWT specifically.

Thirty-one consecutive amateur and professional athletes presenting with GPS were recruited during the study period and included after meeting the predefined eligibility criteria, in order to minimize selection bias. All participants were soccer players, including both amateur and professional athletes, as soccer represents one of the sports most frequently associated with rectus–adductor syndrome and GPS. Professional athletes were defined as individuals who train and compete in soccer as paid work, receiving remuneration for their athletic performance, whereas amateur athletes were defined as individuals for whom soccer participation does not constitute paid employment but rather voluntary competition and physical activity [35]. Due to the prospective, single-arm, single-center pilot study design, causal relationships between the intervention and clinical outcomes cannot be inferred. The diagnosis was established through clinical history, physical examination, and confirmatory imaging with magnetic resonance imaging. Eligible participants were adults aged 18 years to 45 who reported groin pain persisting for at least three months, demonstrated positive clinical tests such as palpation of the pubic insertion of the rectus abdominis/adductor longus aponeurosis and isometric squeeze tests, and had diagnostic confirmation with imaging. Exclusion criteria were previous surgery in the pelvic or groin region, inguinal hernia or other causes of groin pain, bone fracture or stress fracture, recent corticosteroid injection or nerve block, neoplastic disease, acute infection of soft tissues or adjacent bones, pregnancy, coagulation abnormalities, and pacemaker implantation.

### 2.3. Intervention

All participants underwent f-ESWT according to a standardized treatment protocol reflecting the current state of the art in tendinopathy management. Treatments were performed by the principal investigators using the Duolith system (Storz Medical, Tägerwilen, Switzerland), an electromagnetic shockwave generator applied to the target area corresponding to the pubic insertion of the adductor tendon or the rectus abdominis. Patients were treated in a supine position with legs slightly apart to expose the treatment site, and no local anesthesia was used. Each session consisted of 2400 pulses at a frequency of 4 Hz and an energy flux density of 0.20 mJ/mm^2^. Sessions were delivered once weekly for three consecutive weeks [36].

### 2.4. Rehabilitation Program

In addition to f-ESWT, all participants followed a standardized rehabilitation program specifically designed for rectus–adductor syndrome. The rehabilitation program was initiated concomitantly with the first f-ESWT session and continued for a total duration of 16 weeks; therefore, f-ESWT was completed by week 3, while rehabilitation continued for an additional 13 weeks thereafter. Rehabilitation sessions were performed three times per week and supervised by an experienced physical therapist specialized in sports rehabilitation to ensure correct execution and appropriate progression of exercises; adherence was monitored through direct supervision and attendance records. Each session lasted approximately 45–60 min and began with a standardized warm-up including low-intensity aerobic activity and dynamic mobility exercises for the hip and pelvic region. The core component of the program consisted of progressive strengthening exercises targeting the hip adductors (including isometric adduction in supine position, side-lying hip adduction, standing adduction with elastic resistance, and Copenhagen adduction exercises) and abdominal core stabilization exercises. A specific adductor stretching exercise (seated butterfly stretch) was included at the end of each session [37,38]. Exercise intensity and difficulty were progressively increased based on individual tolerance and symptom response, following a pain-monitoring approach allowing mild discomfort without symptom exacerbation to avoid kinesiophobia [21,39,40]. All clinical outcome assessments were conducted on days separate from f-ESWT sessions to minimize the influence of acute treatment-related effects.

### 2.5. Outcome Measures

Outcome measures were assessed at baseline (T0), one month (T1), and four months (T2) after completion of the f-ESWT course. The primary outcome was hip and groin–related function, evaluated using the Hip and Groin Outcome Score (HAGOS), a validated self-administered questionnaire comprising six subscales—Pain, Symptoms, Activities of Daily Living, Sport and Recreation, Participation in Physical Activity, and Quality of Life—with scores ranging from 0 (severe problems) to 100 (no problems) [41].

Secondary outcomes included pain intensity, assessed using the Visual Analog Scale (VAS), which consists of a 100 mm horizontal line anchored by “no pain” (0) and “pain as severe as possible” (10) [42], and the Roles and Maudsley score (RM), used to describe the impact of groin pain on daily activities and overall quality of life, with higher scores indicating poorer outcomes [43].

### 2.6. Data Collection and Follow-Up

All clinical assessments and patient-reported outcome measures were administered at baseline, at 4-week follow-up, and at the end of the 16-week follow-up period by a trained physiatrist experienced in the evaluation of groin pain in athletes. Data were collected prospectively, anonymized, and stored in accordance with institutional data protection procedures. Participant compliance with the rehabilitation program was monitored throughout the study period, and any adverse events or complications related to either f-ESWT or rehabilitation program were systematically recorded during follow-up.

### 2.7. Statistical Analysis

This was an exploratory pilot study, and formal sample size calculation for hypothesis testing was not the primary objective. Considering the primary outcome [44,45], the target enrollment of approximately 30 participants was chosen pragmatically to ensure sufficient precision for descriptive statistics and to assess feasibility. All 31 consecutively recruited athletes with complete datasets were included.

Statistical analysis was performed using SPSS version 28 (IBM Corp, Armonk, NY, USA). Given the repeated-measures design with three time points, a one-way repeated-measures ANOVA was used as the primary analytical approach for each outcome measure, addressing concerns about inflated Type I error from multiple pairwise comparisons. Sphericity was assessed using Mauchly’s test; when violated, Greenhouse-Geisser correction was applied. Post hoc pairwise comparisons were performed using Bonferroni-adjusted paired t-tests. Effect sizes are reported as partial eta-squared (η^2^*p*) for the overall time effect and Cohen’s dz for pairwise comparisons [46,47,48]. Paired-samples *t*-tests with Bonferroni correction were also performed to allow comparison with previous literature using this approach. Statistical significance was set at *p* < 0.05. To contextualize the clinical relevance of the observed changes, results were interpreted against established minimal clinically important difference (MCID) thresholds: 8–12 points for HAGOS in hip and groin conditions [22,44] and 1.5–2.0 points for VAS in musculoskeletal pain [42].

## 3. Results

### 3.1. Patient Demographic and Clinical Characteristics

Thirty-one athletes completed the study and were included in the final analysis. No missing data were observed for any of the outcome measures at baseline (T0), intermediate follow-up (T1), or final follow-up (T2). The descriptive statistics and frequencies of the study sample provide a detailed overview of the participants (Table 1). Among the 31 participants, ages ranged from 19 to 37 years, with a mean age of 28.4 ± 5.8 years, 77.4% were male (24), and 22.6% were female (7). Additionally, 54.8% reported osteitis pubis (17), and 45.2% did not (14), while 45.2% were amateur athletes (14) and 54.8% were professionals (17). Mean weekly training exposure was 12.8 ± 4.2 h for professional athletes and 4.5 ± 1.7 h for amateur athletes.

Repeated-measures ANOVA revealed statistically significant main effects of time for all outcome measures (Table 2).

HAGOS (Primary Outcome): The main effect of time was statistically significant (F[1.6, 48.0] = 241.3, *p* < 0.001, η^2^*p* = 0.89, Greenhouse-Geisser corrected). Mean HAGOSs changed from 47.23 ± 7.79 at T0 to 77.94 ± 16.18 at T1 and 90.00 ± 14.26 at T2. Post hoc comparisons showed significant changes at all intervals: T0 to T1 (mean change: 30.71 points, *p* < 0.001), T0 to T2 (42.77 points, *p* < 0.001), and T1 to T2 (12.06 points, *p* < 0.001). The total change exceeded the MCID (8–12 points) by approximately 3.5–5 times.

VAS Pain: Significant main effect of time (F[1.5, 44.5] = 308.8, *p* < 0.001, η^2^*p* = 0.91, Greenhouse-Geisser corrected). VAS decreased from 6.81 ± 1.25 at T0 to 3.68 ± 1.11 at T1 and 1.90 ± 1.45 at T2. Total reduction: 4.90 points (*p* < 0.001), exceeding MCID by 2.5–3 times.

Roles and Maudsley: Significant main effect of time (F[2, 60] = 77.4, *p* < 0.001, η^2^*p* = 0.72). RM decreased from 2.39 ± 0.50 at T0 to 1.52 ± 0.57 at T1 and 1.26 ± 0.63 at T2.

**Table 2:** Outcome Measures at Baseline and Follow-up.

Outcome	T0 (Baseline)	T1 (1 Month)	T2 (4 Month)	Time Effect *p*	η^2^*p*
**HAGOS**	47.23 ± 7.79	77.94 ± 16.18	90.00 ± 14.26	<0.001	0.89
**VAS (0–10)**	6.81 ± 1.25	3.68 ± 1.11	1.90 ± 1.45	<0.001	0.91
**RM**	2.39 ± 0.50	1.52 ± 0.57	1.26 ± 0.63	<0.001	0.72

Values are mean ± SD. η^2^*p* = partial eta-squared from repeated-measures ANOVA.

The very large effect sizes observed (η^2^*p* > 0.70 for all outcomes) require careful interpretation. Such magnitudes are uncommon in therapeutic studies and likely reflect the combined influence of multiple factors beyond any specific treatment effect, including the natural history of GPS, which often improves over time particularly with activity modification; regression to the mean, as participants were recruited during periods of heightened symptom severity; placebo and contextual effects related to patient expectations, therapeutic attention, and monitoring; and the 16-week structured exercise program, which represents an evidence-based intervention and likely contributed substantially to the observed outcomes. Within this design, the specific contribution of f-ESWT remains unknown and cannot be isolated.

### 3.2. Safety

No serious adverse events were observed. Transient local discomfort during f-ESWT was reported by some participants but did not require treatment discontinuation. All participants completed the three f-ESWT sessions and adhered to the rehabilitation program.

## 4. Discussion

The primary aim of this prospective, single-arm, single-center pilot study was to evaluate short- and medium-term clinical outcomes after f-ESWT combined with a standardized rehabilitation program in athletes with GPS using validated, groin-specific patient-reported and global outcome measures. The main findings were that HAGOS (primary outcome) improved markedly from baseline to both follow-ups, accompanied by substantial reductions in VAS pain and improvements in RM. Improvements were progressive across time points and were associated with large within-subject effect sizes, suggesting clinically relevant symptom and function changes within the observation period.

The magnitude of improvement in HAGOS (mean change of 42.77 points) substantially exceeded the established minimal clinically important difference (MCID) for this instrument, reported as approximately 8–12 points in hip and groin conditions [22,44]. Similarly, the observed reduction in pain intensity, as measured by VAS (mean change of 4.90 points), surpassed the commonly accepted MCID threshold of 1.5–2.0 points for musculoskeletal pain by a factor of two to three times [42]. These changes further support the clinical relevance of the observed outcomes, indicating that patients experienced substantial and meaningful improvements during the treatment period. However, while the improvements exceed established MCID thresholds, in the absence of a control, sham, or comparative treatment group, causal inference remains limited, and the observed improvements cannot be unequivocally attributed to ESWT alone.

Our findings align with previous evidence supporting the use of shockwave therapy in chronic tendinopathies [49,50,51], and similarly, improvement in tendinopathy has been associated with both pain reduction and recovery of mobility in the affected muscle–tendon unit [52,53,54].

In the context of athletic GPS, a randomized controlled trial by Schöberl et al. [33] in football players with osteitis pubis demonstrated an earlier reduction in VAS pain and a faster return to sport in the f-ESWT group compared with controls, highlighting a predominant early-phase effect of f-ESWT, while the progressive improvements observed at medium-term follow-up in the present cohort suggest that f-ESWT, when combined with a standardized rehabilitation program, may also contribute to sustained functional recovery over time.

The only randomized controlled trial currently available on the context of athletic GPS [33] did not report the energy flux density (mJ/mm^2^) applied during ESWT treatment. In the present study, a focused ESWT protocol with an energy flux density of 0.20 mJ/mm^2^ was used. Considering the observational nature of this study and therefore its lower level of evidence compared to a RCT, the comparison is limited. Future randomized controlled studies should therefore aim to investigate potential dose–response relationships by comparing different energy levels, building on evidence from other musculoskeletal conditions in which ESWT protocols with varying energy flux densities have been directly evaluated [23,55,56].

Similarly, the available literature does not clearly define whether f-ESWT alone or combined focused and radial ESWT protocols yield superior clinical outcomes in GPS. While different ESWT modalities may exert distinct biomechanical and biological effects on tendon and peri-tendinous tissues, comparative data in this specific population are lacking. Future randomized studies directly comparing focused ESWT with combined focused and radial approaches are therefore warranted to clarify their relative effectiveness and to inform the development of optimized treatment protocols [57,58,59,60].

Athletic groin pain often follows a prolonged, fluctuating course, with symptoms persisting or recurring without structured load-based rehabilitation. Spontaneous recovery or rest alone is uncommon, particularly in high-demand athletes, highlighting the need for targeted therapeutic interventions [21,22,61].

In the treatment of musculoskeletal pain, increasing attention has been directed toward combined therapeutic strategies that integrate physical modalities with active rehabilitation, with the aim of addressing both pain reduction and restoration of tissue load capacity [22,31,37,62,63,64]. In this context, exercise-based rehabilitation has demonstrated beneficial effects on pain and function in athletes with GPS, particularly through targeted adductor strengthening and progressive load management. Randomized trials have shown that active exercise therapy is superior to passive interventions in long-standing adductor-related groin pain. In the main randomized studies on long-standing adductor-related groin pain, the amount of supervised physiotherapy varied considerably. In Hölmich 1999, the protocol consisted of three sessions per week over 8–12 weeks, with a median of approximately 15 supervised sessions actually delivered [21]. In contrast, in Weir 2011, the exercise therapy group received only three supervised sessions combined with a home-based program, while the multimodal manual therapy group underwent just one to two treatment sessions [22]. In our study, patients underwent a substantially higher treatment volume, completing three supervised sessions per week for 16 weeks, resulting in a markedly greater cumulative rehabilitation load compared with previous papers. Building on this evidence, the present study adopted a combined approach integrating f-ESWT within a structured exercise-based rehabilitation program to support both symptom modulation and functional recovery.

Exercise-based rehabilitation, has demonstrated beneficial effects on pain and function in athletes with groin pain. Randomized trials have shown that active exercise therapy is superior to passive or minimal interventions in long-standing adductor-related groin pain, leading to higher rates of clinical improvement. Systematic reviews further support exercise therapy as a cornerstone of conservative management, although variability in protocols and outcomes remains [21,22,65]. Adequately powered RCTs comparing f-ESWT plus rehabilitation versus rehabilitation alone are essential to isolate the specific contribution of f-ESWT.

Placebo effects may play a relevant role in the management of muskuloskeletal pain. Although sham-controlled trials specifically targeting groin pain are scarce, a study on patellar tendinopathy and ESWT have shown meaningful pain reductions in placebo or sham groups, highlighting the influence of contextual and expectancy-related factors [66].

In the absence of a large body of evidence specifically addressing ESWT in GPS, its use can be contextualized within the broader literature on tendinopathies. Focused and radial ESWT have demonstrated beneficial effects on pain and function in several chronic tendon disorders, including rotator cuff tendinopathy [36,43,49], lateral epicondylitis [60,67,68], and Achilles tendinopathy [51,69,70]. These effects have been attributed to a combination of analgesic mechanisms, modulation of tendon remodeling, and improved tissue load tolerance. Such consistent findings across different anatomical regions support the biological plausibility of ESWT as an adjunctive treatment in load-related groin tendon disorders [71].

Future research should build upon these preliminary findings by addressing several key priorities. Adequately powered randomized controlled trials comparing focused extracorporeal shock wave therapy (f-ESWT) combined with rehabilitation versus rehabilitation alone are necessary to clearly isolate the specific contribution of f-ESWT to clinical outcomes. In addition, dose–response studies examining different energy flux densities, numbers of sessions, and treatment frequencies would help optimize treatment protocols and improve standardization. Comparative effectiveness research is also warranted, particularly trials evaluating focused versus radial ESWT or ESWT against other adjunctive interventions such as intratissue percutaneous electrolysis, in order to better inform clinical decision-making. Longer-term follow-up studies extending to 12–24 months are essential to determine the durability of benefits and to assess recurrence rates. Finally, data-driven approaches, including the application of machine learning techniques, may facilitate the identification of patient subgroups most likely to respond to specific therapeutic combinations, thereby supporting more personalized rehabilitation strategies. Groin pain syndrome encompasses a broad spectrum of clinical presentations affecting the inguinal–pubic–adductor region and interfering with sports and daily activities, reflecting a highly heterogeneous and multifactorial condition. Given this complexity, future research may benefit from data-driven approaches, including artificial intelligence models, to integrate clinical, imaging, and comorbidity-related variables in order to support treatment stratification and optimize therapeutic decision-making [72,73,74,75,76].

## 5. Strengths and Weaknesses of the Study

Several strengths and limitations of this study warrant consideration. A key strength lies in the use of the HAGOS questionnaire, a groin-specific, validated patient-reported outcome measure that enhances the sensitivity and clinical relevance of the results. Additionally, the standardized and detailed rehabilitation protocol ensured consistency in adjunctive care, minimizing variability in non-intervention components. Consecutive patient enrollment further strengthens the external validity of the findings by reducing selection bias.

Despite these strengths, several limitations must be acknowledged. The absence of a control or placebo group limits the ability to draw causal inferences about the observed improvements. Furthermore, the lack of blinding of participants and clinicians introduces the potential for performance and detection bias. The single-center design may also restrict the generalizability of findings across different clinical settings. Additionally, the observed improvements could be influenced by regression to the mean, particularly given that participants were recruited during periods of heightened symptom severity. Finally, the follow-up period was limited to four months; longer-term data are needed to evaluate the durability of response and potential recurrence rates.

Despite these limitations, this study contributes meaningful clinical evidence supporting the integration of focused shockwave therapy in the conservative management of adductor-related groin pain. Future randomized controlled trials with larger cohorts and longer follow-ups are warranted to confirm these preliminary findings and refine patient selection criteria.

## 6. Conclusions

In this prospective single-arm pilot study, a multimodal conservative program combining focused ESWT (3 weekly sessions) and structured rehabilitation (16 weeks) was associated with temporal changes in pain and groin-specific function that substantially exceeded established MCID thresholds in athletes with GPS. The intervention was well-tolerated with no serious adverse events.

These findings are preliminary and purely descriptive. The single-arm design and absence of a control group preclude any causal inferences regarding treatment effectiveness. The observed changes cannot be attributed to f-ESWT specifically, as the concurrent 16-week rehabilitation program—an intervention with established efficacy in GPS—likely contributed substantially to the outcomes. The very large effect sizes observed further suggest the influence of non-specific factors including natural history, regression to the mean, and placebo effects.

These hypothesis-generating observations may inform the design of future controlled trials. Adequately powered randomized studies comparing f-ESWT plus rehabilitation versus rehabilitation alone are essential before any conclusions about f-ESWT effectiveness in GPS can be drawn.

## Figures and Tables

**Table 1:** Baseline variables and comparison of outcomes measures before and after treatment.

Characteristic	Value
Participants	31
Gender	Male: 77.4%; Female: 22.6%
Osteitis Pubis	Yes: 54.8%; No: 45.2%
Athletic Status	Amateur: 45.2%; Professional: 54.8%
Age (years)	Mean: 28.4 ± 5.8; Range: 19–37
Weekly training, (hours)	Amateur: 4.5 ± 1.7; Professional: 12.8 ± 4.2

Values are reported as mean ± standard deviation for continuous variable and as distribution for categoric variable and *p* value.

## Data Availability

The data presented in this study are available on request from the corresponding authors due to privacy and ethical restrictions related to the protection of participant data.
